# Endoscopic endonasal transclival removal of tumors of the clivus and anterior region of the posterior cranial fossa (results of surgical treatment of 140 patients)

**DOI:** 10.1186/s41016-018-0144-5

**Published:** 2018-11-15

**Authors:** Alexey N. Shkarubo, Konstantin V. Koval, Ilia V. Chernov, Dmitry N. Andreev, Alexey B. Kurnosov, Andrey A. Panteleyev

**Affiliations:** Federal State Autonomous Institution, N.N. Burdenko National Medical Research Center of Neurosurgery of the Ministry of Health of the Russian Federation (N.N. Burdenko NMRCN), 4-ya Tverskaya-YAmskaya street, 16, Moscow, Russian Federation

**Keywords:** Clivus, Clival chordoma, Endoscopic endonasal transclival approach, Monitoring of cranial nerves, Posterior cranial fossa, Skull base anatomy, Skull base surgery

## Abstract

**Background:**

Until recently, tumors of the clivus and the anterior region of the posterior cranial fossa were considered extremely difficult to access and often inoperable using standard transcranial approaches. With the introduction into the neurosurgical practice of minimally invasive methods utilizing endoscopic techniques, it became possible to effectively remove hard-to-reach tumors, including central tumors of the anterior region of the posterior cranial fossa.

**Methods:**

From 2008 to the present time, the inpatient institution has operated on 140 patients with various tumors of the base of the skull, localized to the clivus and anterior region of the posterior cranial fossa (65 men and 75 women). The age of patients ranged from 3 to 74 years. Tumor distribution according to the histopathological features was as follows: chordomas, 103 (73.57%); meningiomas, 12 (8.57%); pituitary adenomas, 9 (6.43%); fibrous dysplasia, 4 (2.86%); cholesteatoma, 3 (2.14%); craniopharyngiomas, 2 (1.43%); plasmacytomas, 2 (1.43%); and other tumors (giant cell tumor, neurohypophyseal glioma, osteoma, carcinoid, chondroma), 5 (3.57%). The tumors had the following size distribution: giant (more than 60 mm), 35 (25%); large (35–59 mm), 83 (59.3%); medium (21–35 mm), 21 (15%); and small (less than 20 mm), 1 (0.7%). In 11 cases, intraoperative monitoring of the cranial nerves was performed (21 cranial nerves were identified).

**Results:**

Upper, middle, and lower transclival approaches provide access to the anterior surface of the upper, middle, and lower neurovascular complexes of the posterior cranial fossa. The chordoma cases were distributed as follows according to extent of removal: total removal, 68 (66.02%); subtotal removal, 25 (24.27%); and partial removal, 10 (9.71%). The adenomas of the pituitary gland were removed totally in 6 cases, subtotally in 1 case and partially in 2 cases. The meningiomas were removed totally in 1 case, subtotally in 5 cases, and partially in 5 cases, with less than 50% of the tumor removed in 1 case. Other tumors (cholesteatoma, craniopharyngioma, fibrous dysplasia, giant cell tumor, glioma of the neurohypophysis, osteoma, plasmacytoma, carcinoid, and chondroma) were removed totally in 9 cases and subtotally in 7 cases. Postoperative CSF leaks occurred in 9 cases (6.43%) and meningitis in 13 cases (9.29%). Oculomotor disorders developed in 19 patients (13.57%), 12 of which regressed during the period from 4 to 38 days after surgery, and 7 of which were permanent. In 2 cases, surgical treatment had a lethal outcome (1.43%).

**Conclusion:**

The endoscopic endonasal transclival approach can be used to obtain access to the centrally located tumors of the posterior cranial fossa. It is an alternative to transcranial approaches in the surgical treatment of tumors of the clivus. The results of using this approach are comparable with the results of transcranial and transfacial approaches and, in some cases, surpass them in effectiveness. The extended endoscopic endonasal posterior (transclival) approach, considering its minimally invasive nature, allows fora radical and low-risk (in terms of postoperative complications and lethality) removal of various skull base tumors of central localization with the involvement and without the involvement of the clivus, which, until recently, were considered to be almost inoperable.

## Background

The region of the posterior cranial fossa, including the clivus and the anterior surface of the brainstem, is considered the hardest-to-access region in skull base surgery.

Despite the rapid development of various surgical techniques over the past decade, the treatment of tumors of the clivus and the surrounding anatomical structures is still a daunting task for a neurosurgeon.

Surgical interventions involving the clivus, as well as other areas of the base of the skull, are associated with a number of limiting factors: significant depth of the surgical wound with a complex anatomical environment, including the major blood vessels and cranial nerves, and the effect of the tumor on the structures of the brain stem.

Recently, significant progress has been made in the development of endoscopic endonasal surgery, which has already surpassed some transcranial and transfacial approaches in the treatment of tumors of the clivus. The endoscopic endonasal transclival approach to the structures of the posterior cranial fossa allows to effectively overcome some of the limiting factors mentioned above. It provides a direct view of the median structures of the skull base without applying traction to various structures of the brain [1–4].

A well-known fact that characterizes the advantages of the endoscopic transnasal approach is that it can be used to access almost the entire base of the skull and the craniovertebral transition, from the posterior ethmoidal air cells to the axis, and allow for radical tumor removal with minimal impact on the stem structures. Various types of anterior approaches to the clivus are used for the removal of intradural tumors of the base of the skull, most of which were described in the 1970s and 1980s [5]. Currently, there is a fairly large number of options for the anterior approach to the clivus (transoral, transsphenoidal, transmaxillary, transfacial, transbasal, transtemporal). Some of these approaches require extensive resection of the facial, cranial, oral, and nasal structures [5–9]. Couldwell et al. [6] demonstrate the advantages and shortcomings of each of these approaches in their work.

With the development of modern endoscopic techniques, more and more studies have started to emerge in the world literature regarding the experience of surgical removal of centrally located tumors of the base of the skull using the endoscopic transclival approach [10–20]. According to Sanmillan et al. [15], the results of using the endoscopic transclival approach for the removal of central tumors of the skull base are comparable to the results of using various transcranial approaches, and in a number of cases, they are clearly superior.

For the removal of tumors located along the midline of the clivus and above the hard palate (extra- or intradural), it is advisable to use the extended posterior endoscopic endonasal approach.

Our experience of surgical treatment of various tumors of the skull base, localized in the region of the clivus and the anterior region of the posterior cranial fossa (chordomas, pituitary adenomas, meningiomas, cholesteatomas, etc.) using the endoscopic transclival approach, totals 140 patients. According to the leading experts in the field, the number of such surgeries to date is as follows: Aldo Stamm [10], 23 surgeries; Vellutini Ede et al. [12], 38 surgeries; Tamura et al. [13], 24 surgeries; Al-Mefty et al. [14], 43 surgeries; Koutourousiou et al. [11], 60 surgeries; and Sen et al. [21], 71 surgeries.

## Methods

**Fig. 1:**
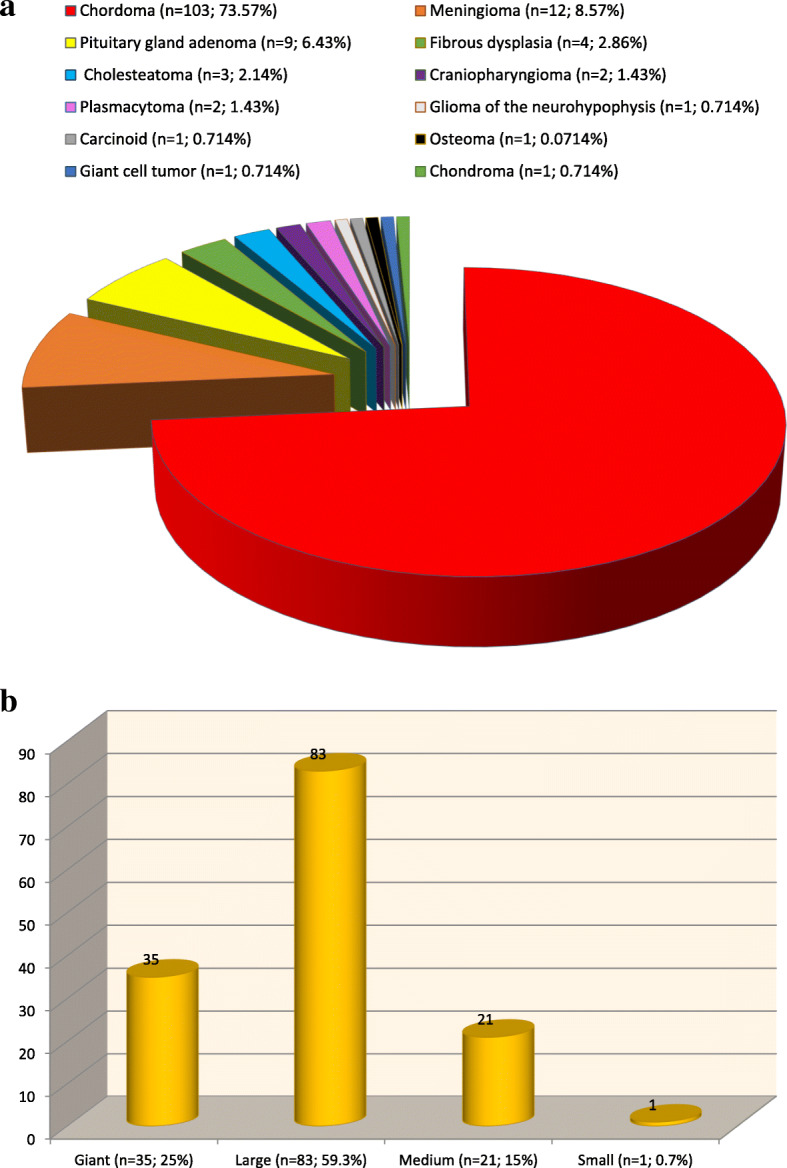
**a** Distribution of tumors according to histological structure (*n* = 140). **b** Distribution of tumors according to size (*n* = 140). Horizontal coordinates—tumor size; vertical coordinates—number of cases

Personal surgical experience of the first author is 140 patients. The distribution of the tumors according to the histological structure is presented in Fig. 1a. The distribution of tumor dimensions was as follows (Fig. 1b): giant (greater than 60 mm), 35 (25%); large (35–59 mm), 83 (59.3%); medium (21–35 mm), 21 (15%); and small (less than 20 mm), 1 (0.714%).

Data on the distribution of tumor localizations (different sections of the clivus) and extension to adjacent anatomical regions is presented in Tables 1 and 2.

**Table 1:** Localization of tumors by section of the clivus

Clivus section	Number of cases	Percent
Upper	13	9.29
Middle	6	4.29
Lower	1	0.7
Upper and middle	68	48.57
Middle and lower	11	7.86
Whole clivus (upper, middle, lower)	41	29.29
Total	140	100

**Table 2:** Extension of tumors from the clival region into the adjacent anatomical regions

Zone of adjacent extension of the tumor	Number of cases	Percent
Cavernous sinus (unilateral extension)	25	17.9
Turkish saddle	13	9.3
Parasellar region (bilateral extension into the cavernous sinuses)	12	8.6
Suprasellar region	20	14.3
Apex of the temporal bone pyramid	9	6.4
Cerebellopontine angle	8	5.7
Sphenoidal sinus	27	19.3
Nasopharynx	7	5.0
Oropharynx	3	2.1
Posterior ethmoidal air cells	4	2.9
Orbit region	2	1.4
Inner opening of the auditory tube	3	2.1
Temporal lobe, subcortical structures	4	2.9

All patients underwent endoscopic endonasal transclival removal of tumors located in the central region of the clivus and extending into the posterior cranial fossa. Patients also underwent clinical, ophthalmological, neurological, and endocrinological examinations. Before surgery, all patients underwent high-resolution CT with three different views. To determine the relationship of the tumor with the major blood vessels, MR imaging was performed with intravenous contrast and without intravenous contrast enhancement, MR angiography, and SCT angiography. In ten patients, we used intraoperative neuromonitoring of the cranial nerves using a technique developed by us [22–24]. We used the Karnofsky Scale for evaluating the health status of the patients. In the early postoperative period, CT and/or MRI studies were performed to control the radicality of tumor removal. The extent of tumor removal was determined based on follow-up MRI and CT with contrast enhancement data, as well as the intraoperative endoscopic findings, which, in some cases, allowed to visualize the remaining tumor fragments not seen on MRI or CT.

When the bone of the clivus was intact, trepanation was carried out strictly within the limits, required for the safe removal of the tumor. In case of partial or complete destruction of the bony structure of the clivus, resection of the pathological bone tissue was performed until the visually intact bone was observed. The removal of chordomas and other tumors was considered radical when no tumor tissue was identified on control MRI or CT with contrast enhancement.

The radicality of tumor removal was evaluated according to the scale proposed by Frank and Pasquini [25]:

- Radical or total removal—no evidence of tumor tissue on postoperative CT and/or MRI;
- Subtotal removal—volume of the remaining tumor tissue is less than 20% of the original size of the tumor;
- Partial removal—volume of the remaining tumor tissue is less than 50% of the original size of the tumor;
- Insufficient removal—volume of the remaining tumor tissue is more than 50% the original size of the tumor.

**Fig. 2:**
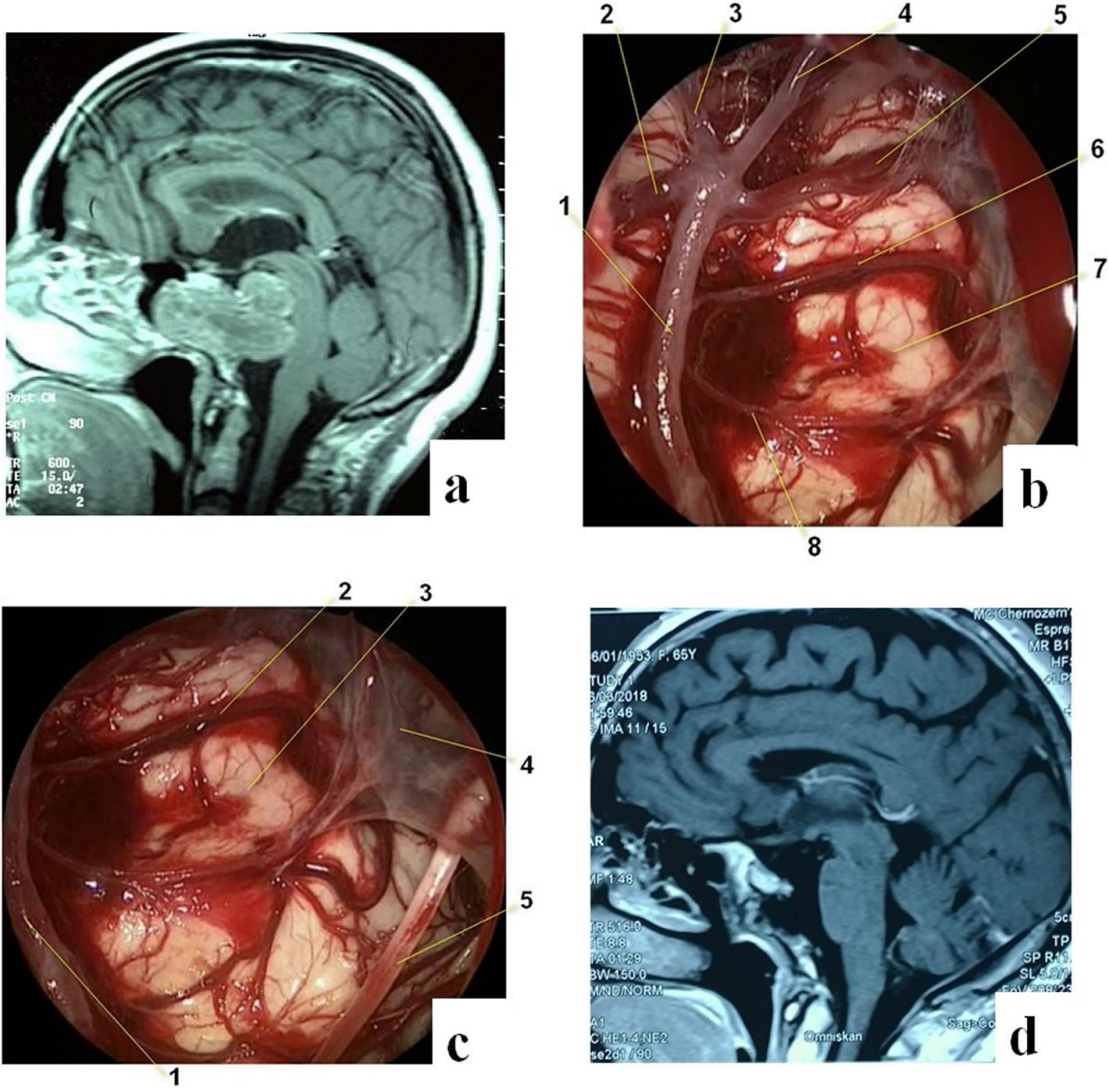
Clinical case 1. 57-year-old female patient with a large skull base chordoma, leading to severe brainstem compression. **a** Preoperative MRI. **b** Intraoperative photograph: 1—basilar artery; 2— right SCA; 3—right PCA; 4—left PCA; 5—left SCA; 6—left AICA; 7—lower pons; and 8—left PICA. **c** Intraoperative photograph: 1—basilar artery; 2—left AICA; 3—upper pons; 4—arachnoid mater; and 5—left abducent nerve. **d** MRI 7.5 years after surgery (no signs of tumor recurrence)

When removing cholesteatomas, the resection was considered radical when the tumor was removed together with its capsule. However, given the nature of the extension of these tumors, as well as abundant adhesions, which are often observed due to aseptic inflammation, complete removal of the capsule is not always possible, especially considering that one patient was previously operated transcranially three times. In patients with fibrous dysplasia, the radicality of removal was assessed according to the boundaries formed by visually intact bone, as well as postoperative CT. In patients with pituitary adenomas, the radicality was assessed using MRI with contrast enhancement. In our study, the number of patients with pituitary adenomas was nine, among them hormonally active adenomas totaled five (four prolactinomas and one ACTH-secreting adenoma). In the postoperative period, complete remission was observed in all prolactinoma cases; however, the case with the ACTH-secreting adenoma had a lethal outcome. Dissection of the dura mater is usually carried out via a linear cut. Figure 2 (case 1) demonstrates the general view of the surgical field during trepanation of the upper, middle, and lower sections of the clivus after removal of a large tumor, the structures of the brain stem, and the major vessels located in the respective sections of the clivus. When approaching the apex of the temporal bone pyramid from the lateral side, it is advisable to use a navigation system, due to the close proximity of the petrosal segments of the internal carotid arteries. Removal of intradural and extradural tumors is accomplished using special vacuum aspirators, curettes, and rongeurs. Bleeding from the cavernous sinus or basilar venous plexus, and often from both, can be controlled using hemostatic agents. If the tumor is intradural, its removal requires the dissection of the dura mater, sometimes, however, the dura can already be destroyed by the tumor (often seen with chordomas). In solid tumors (chordoma, meningioma, etc.), it is necessary to use an ultrasound ablation device. It is imperative to preserve small vascular branches extending from the major vessels (basilar artery, posterior cerebral, and vertebral arteries). If it is impossible to separate a very dense fragment of a tumor from a major vessel, it is advisable to leave this part of the tumor intact in order to avoid damage to the vessel, which could lead to life-threatening bleeding or ischemia of the brainstem structures. The remaining portion of the tumor fused with the major vessel can later be subjected to radiation therapy. For visualization purposes, during different stages of surgical intervention, various endoscopes can be successively used (0°, 30°, 45°, or 70°). In cases when tumor removal follows dissection of the dura mater, duraplasty and skull base defect reconstruction should be carried out using a flap of the fascia lata, adipose tissue of the thigh, bone and cartilage of the nasal septum, and fibrin glue. In a number of the described cases, reconstruction of the dura mater was carried out using our original patented microsurgical technique described elsewhere [26, 27].

**Fig. 3:**
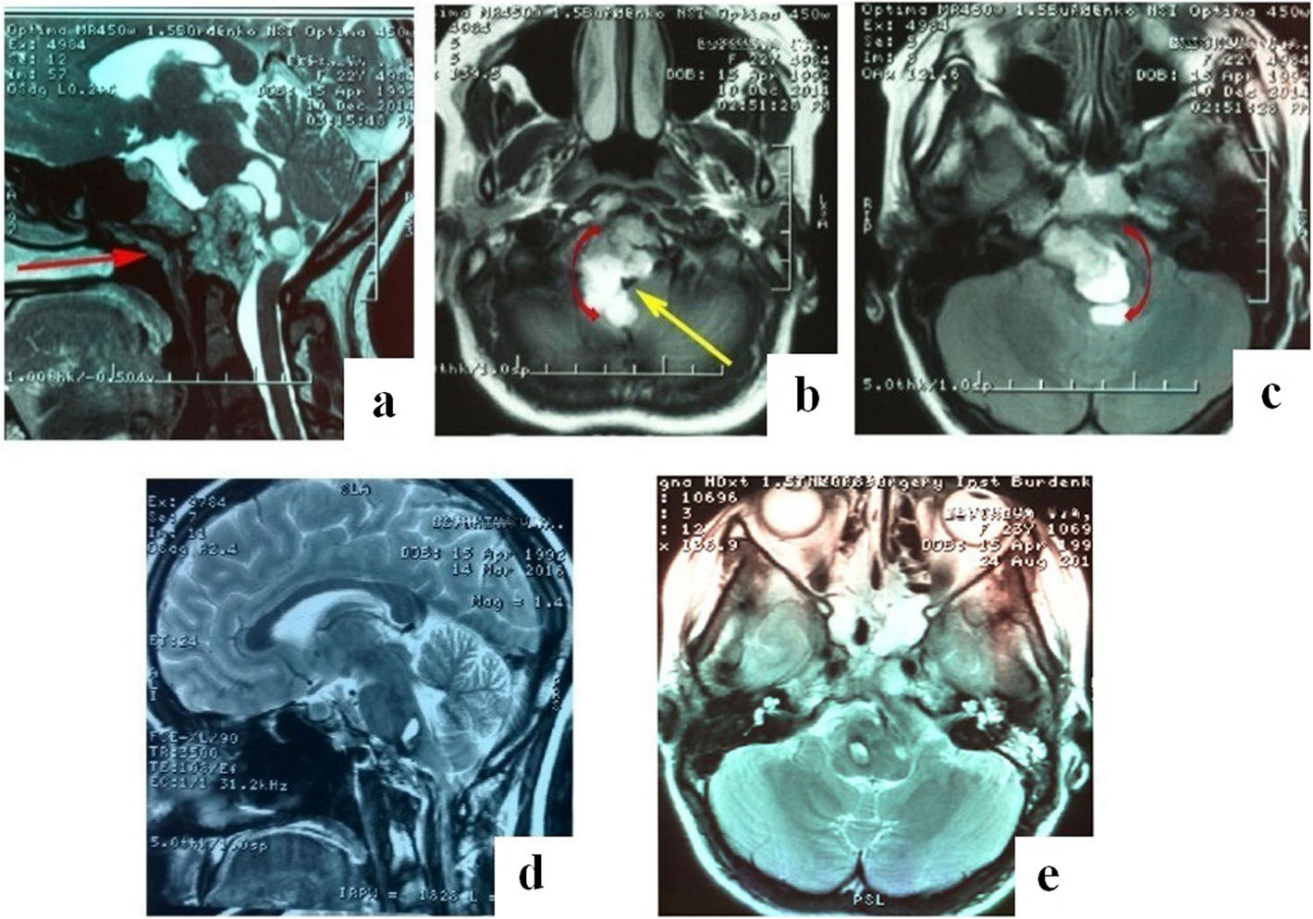
Case 2. Female patient, 22 years old, with giant chordoma of the skull base, with severe compression of the medulla oblongata and midbrain. **a**–**c** Preoperative MRI images: **a** Straight red arrow indicates the direction of transnasal approach. **b**, **c** Curved red arrows indicate the direction of approach to the posterior portion of the tumor around the brain stem structures. **b** Yellow arrow indicates basilar artery location. **d**, **e** MRI images 12 months after surgery, subtotal tumor resection

In case 2 (Fig. 3), the giant chordoma with brain stem compression with a complex relationship of the tumor with the basilar artery was removed subtotally due to significant intraoperative hemodynamic changes, likely caused by the brainstem reacting to the intervention. In light of the high risk of complications, further tumor resection was halted.

**Fig. 4:**
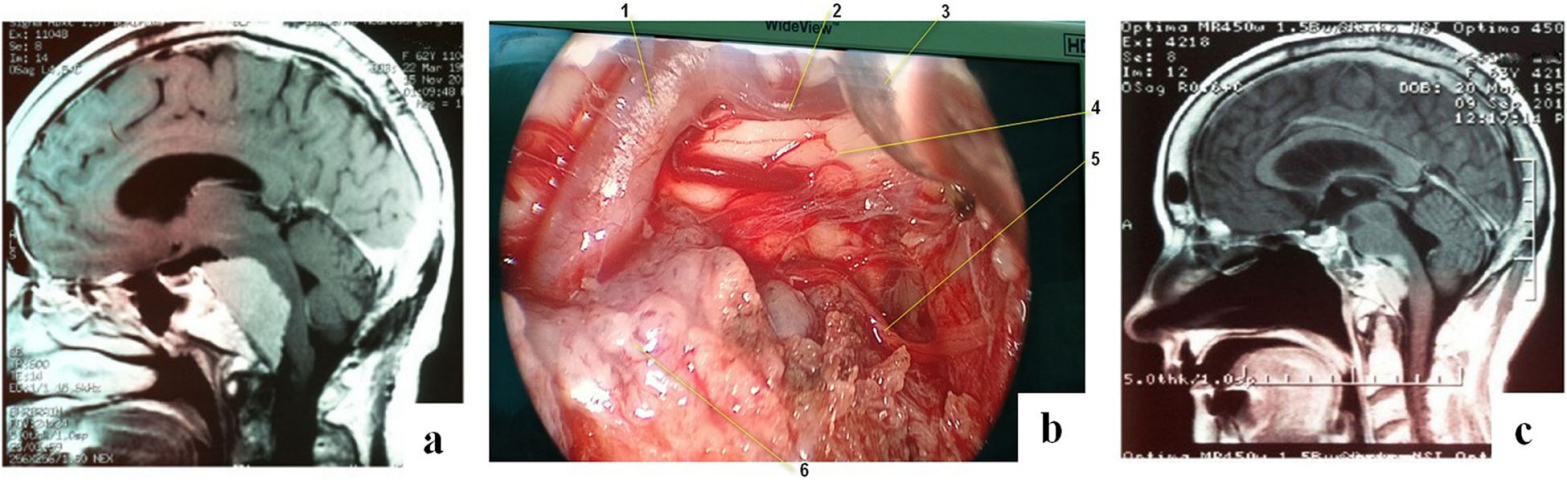
Case 3. Sixty-three-year-old female patient with a giant meningioma of the clivus and the left cerebellopontine angle. **a** MRI images before surgery. **b** Intraoperative photograph: 1—basilar artery; 2—left SCA; 3—aspirator; 4—upper pons; 5—left abducent nerve; and 6—tumor mass. **c** MRI images 4 months after surgery, subtotal removal of the tumor

In case 3 (Fig. 4) with large clival meningioma, the total removal of the tumor was not technically feasible due to the very intimate fusion of its lower portion with the basilar artery.

**Fig. 5:**
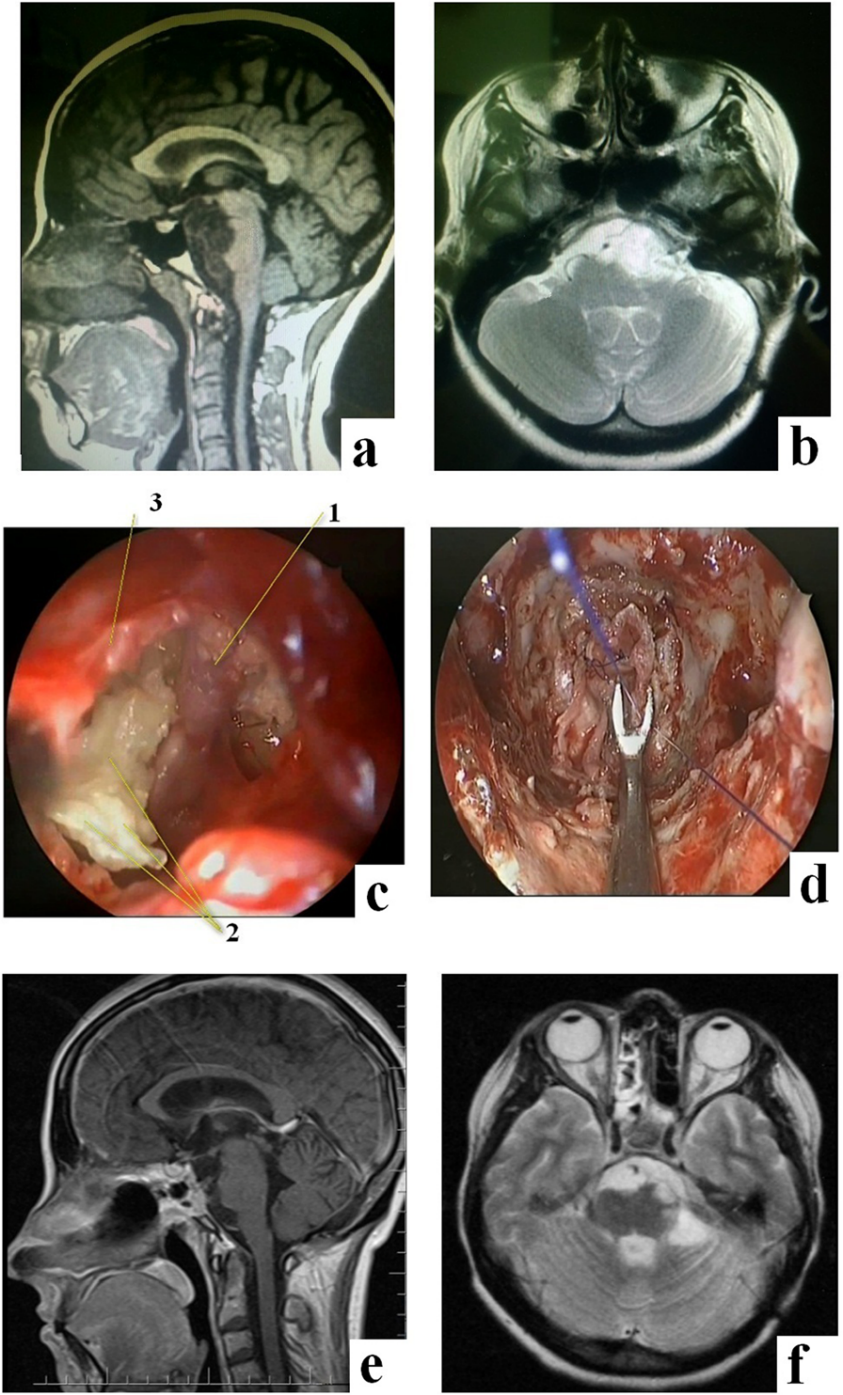
Thirty-four-year-old female patient, with large cholesteatoma of the posterior cranial fossa. **a**, **b** MRI before surgery. **c** Intraoperative photograph: 1—basilar artery; 2—cholesteatoma mass; and 3—dura mater of the clival region. **d** Intraoperative photograph at the stage of skull base defect reconstruction with microsutures. **e**, **f** MRI 4 months after surgery

Figure 5 shows a clinical case of large cholesteatoma, roughly compressing the brainstem structures. Skull base reconstruction was performed using fascia lata autograft with microsutures.

## Results

Evaluation of the clinical dynamics of the disease before and after surgery is presented in Table 3.

**Table 3:** Clinical symptoms before surgery and postoperative symptom dynamics

Type of disorder	Number of cases (%)	Regression/normalization (%)	No changes (%)	Deterioration (%)
Oculomotor disorders (CNs III, IV, VI)	90 (64.28%)	69 (76.7%)	14 (15.5%)	7 (7.8%)
Impaired function of CN V	44 (31.4%)	42 (95.45%)	2 (4.55%)	–
Visual impairment	24 (17.1%)	18	6	–
Bulbar disorders (dysphagia/dysphonia)	38 (27.1%)	32 (84.21%)	4 (10.53%)	2 (5.26%)
Hemiparesis	12 (8.57%)	12	–	–
Hearing impairment	15 (10.7%)	13	2	–
Facial asymmetry (paresis of CN VII)	11 (7.86%)	11	–	–
Coordination disorders	25 (17.86%)	25	–	–
Brainstem symptoms	10 (7.14%)	10	–	–
Cerebral symptoms (cephalgia)	58 (41.42%)	58 (100%)	–	–
Endocrine disorders	8 (5.7%)	5	–	3
Nasal breathing disorders	5 (3.57%)	5	–	–
Hydrocephalus (papilledema)	8 (5.7%)	8	–	–
Conductive sensory disorders	5 (3.57%)	5	–	–
Cognitive disorders (mental disorders)	10 (7.14%)	9	–	1
Arterial hypertension	4 (2.86%)	4	–	–

The extent of tumor removal was evaluated according to the scale proposed by Frank and Pasquini [25] (Table 4). Data regarding the general extent of tumor removal, as well as the extent of tumor removal according to their histologic structure (chordoma, pituitary adenoma, meningioma, cholesteatoma, fibrous dysplasia, craniopharyngioma) is presented in Tables 4 and 5.

**Table 4:** Total resection of tumors (in accordance with the scale by Frank, 2002)

Extent of tumor removal	Number of cases	Percent
Total removal	84	60
Subtotal removal	37	26.43
Partial removal	18	12.86
Insufficient removal	1	0.71

**Table 5:** Extent of tumor removal according to histological structure

Extent of tumor removal	Number of cases
Chordomas (*n*_gen_ = 103)	
Total	68
Subtotal	25
Partial	10
Meningiomas (*n*_gen_ = 12)	
Total	1
Subtotal	5
Partial	5
Insufficient	1
Pituitary adenomas (*n*_gen_ = 9)	
Total	6
Subtotal	1
Partial	2
Fibrous dysplasia (*n*_gen_ = 4)	
Total	2
Subtotal	2
Cholesteatomas (*n*_gen_ = 3)	
Total	1
Subtotal	2
Craniopharyngiomas (*n*_gen_ = 2)	
Total	1
Subtotal	1

Following is the extent of the removal of the remaining tumors (*n* = 5): giant cell tumor—subtotal; osteoma—total; carcinoid—subtotal; and glioma of the neurohypophysis—total; chondroma—total. Plasmacytomas (*n* = 2) were removed totally. The extent of removal of different tumors is demonstrated via specific cases shown in Figs. 2, 3, 4, and 5.

The main complications of the postoperative period (Table 6) were oculomotor disorders, the most common of which included dysfunction of the abducent nerve (15 cases—in 9 cases, functional disorders regressed over a period from 1 to 4 months after surgery, and in 6 patients, permanent neurological deficit was observed); oculomotor nerve paresis (observed in 4 patients—2 of which marked symptomatic regression was observed over a period from 2 to 3 months after surgery, and 2 patients had permanent deficit).

**Table 6:** Postoperative complications

Type of complication	Number of cases	Percent
Cranial nerve dysfunction^a^		
III	4	2.86
VI	15	10.71
IX, X	1	0.71
CSF leakage (without meningitis)	1	0.71
CSF leakage + meningitis	8	5.71
Meningitis (without CSF leakage)	5	3.57
Hydrocephalus	2	1.43
Mental disorders	2	1.43
Death	2	1.43

^a^Primary CN function deficit after surgery. Regression CN dysfunction was observed in ten patients over the period from 1 to 4 months after surgery

Cerebrospinal fluid (CSF) leakage was noted in 9 cases (6.42%), meningitis in 5 cases (3.57%) (the duration of the surgery ranged from 210 to 510 min with an average of 380 min and median of 360 min), and a combination of CSF leakage and meningitis in 8 cases (5.71%) (the duration of the surgery ranged from 150 to 570 min with an average of 364 min and median of 340 min). In total, the duration of surgery in all patients (*n* = 140) ranged from 90 to 570 min (average 271 min, median 255 min). To stop the CSF leakage, CSF fistula surgeries were performed in all 9 patients; a total of 11 surgeries were performed (2 patients were operated twice).

We utilized an antibiotic prophylaxis regimen developed at our clinic: 1 h before surgery, 1.2 g of аmoxicillin/clavulanic acid is administered, with two more doses administered 3 and 6 h later. In cases of meningitis, a 2-g dose of meropenem is administered three times daily and 2 g of vancomycin daily for 7 days. The therapy is continued as needed.

**Fig. 6:**
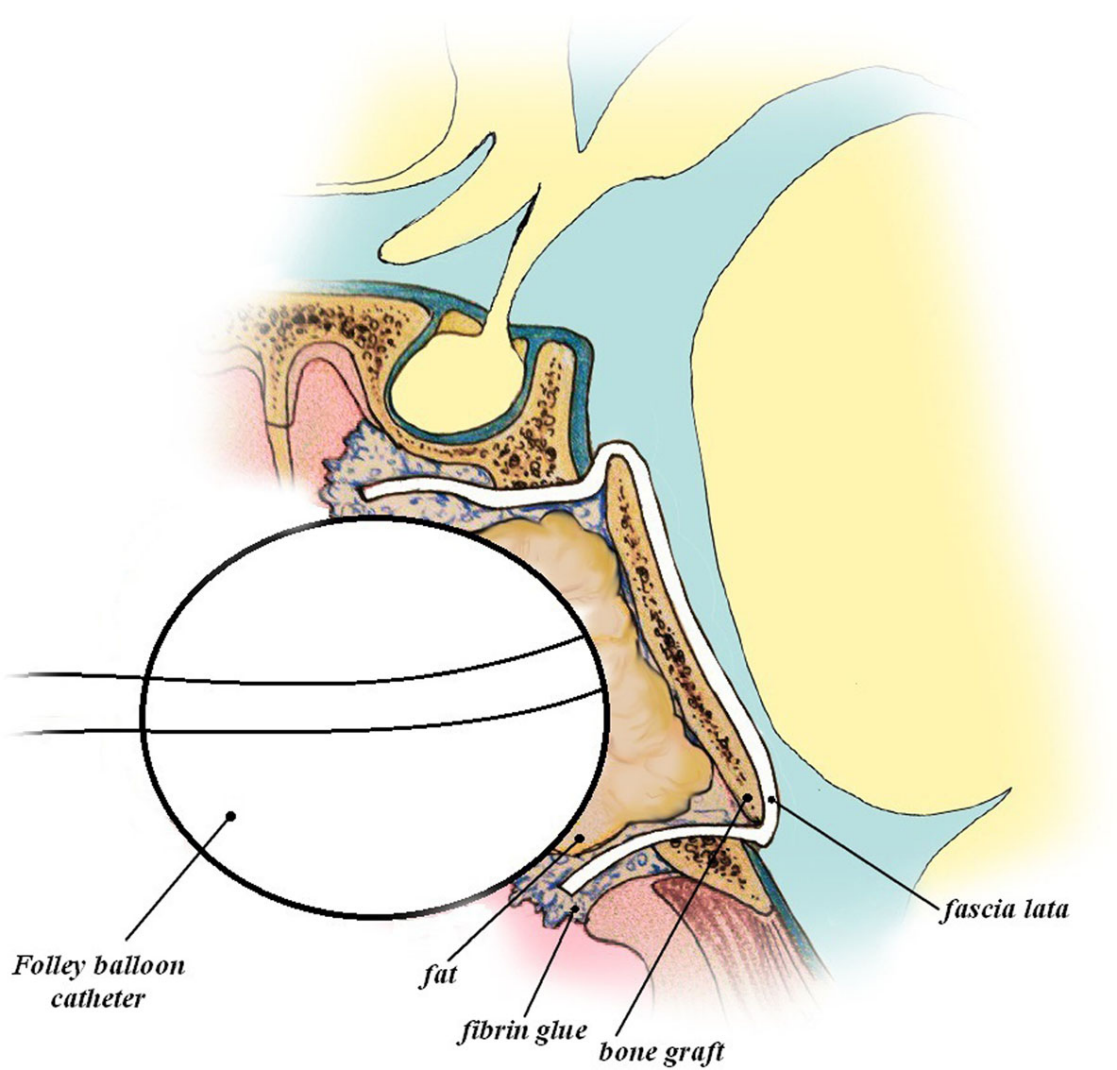
The gasket-seal skull base defect reconstruction technique

Three skull base reconstruction techniques were used in our study: the gasket-seal (fascia lata, bone, fibrin glue) (Fig. 6), the double-needle microsuture technique (suture diameter of 5-0, 6-0, and 4-0). In cases when the dura mater edges could be approximated, they were fixed using microsutures.

**Fig. 7:**
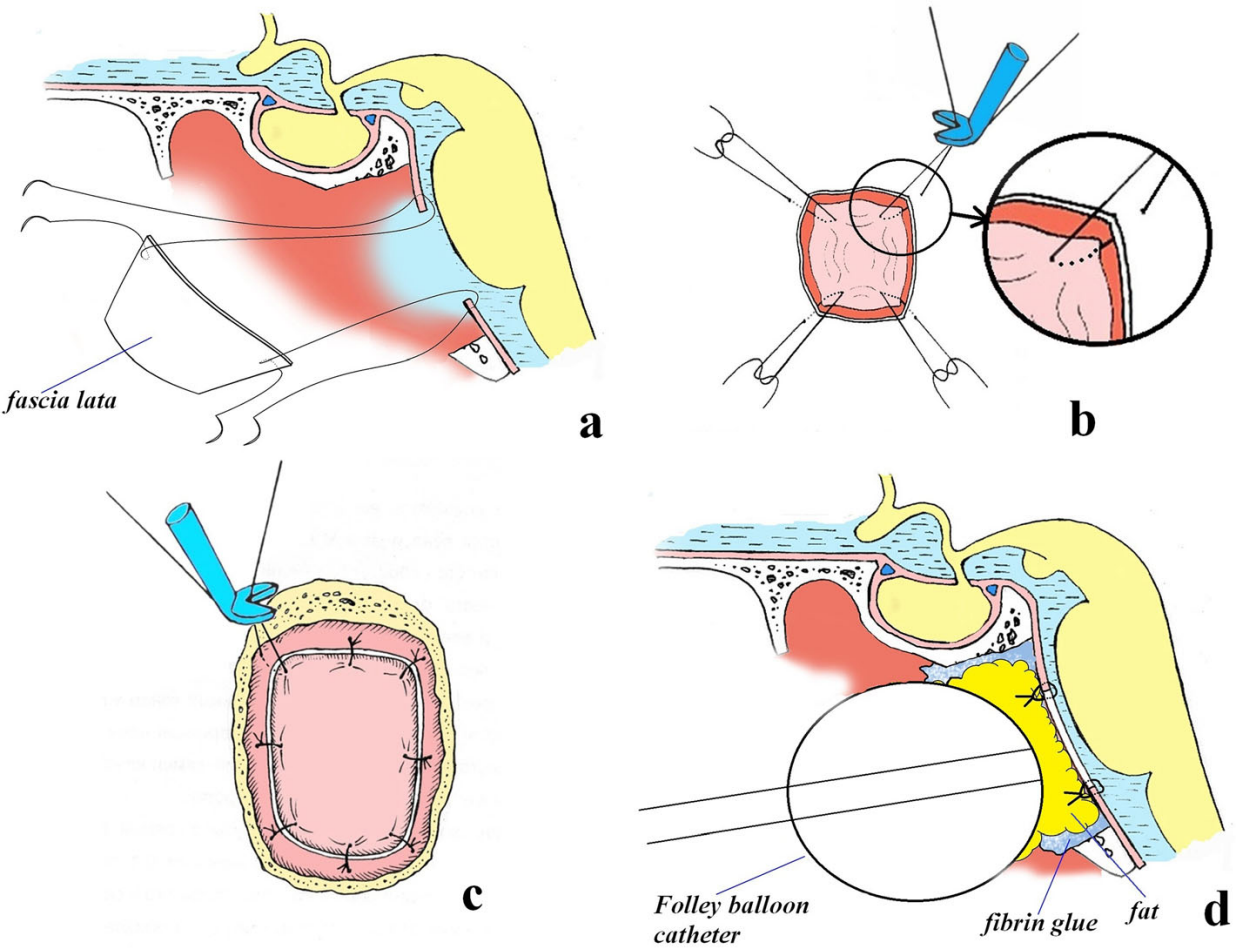
Skull base reconstruction technique using fascia lata, fixed to the dura mater with microsutures along the defect perimeter. **a** Initial reconstruction stage. **b** Fixation of the free autograft at the corners and its lowering into the dura mater defect. **c** Microsutures along the autograft perimeter. **d** Appearance after reconstruction

**Fig. 8:**
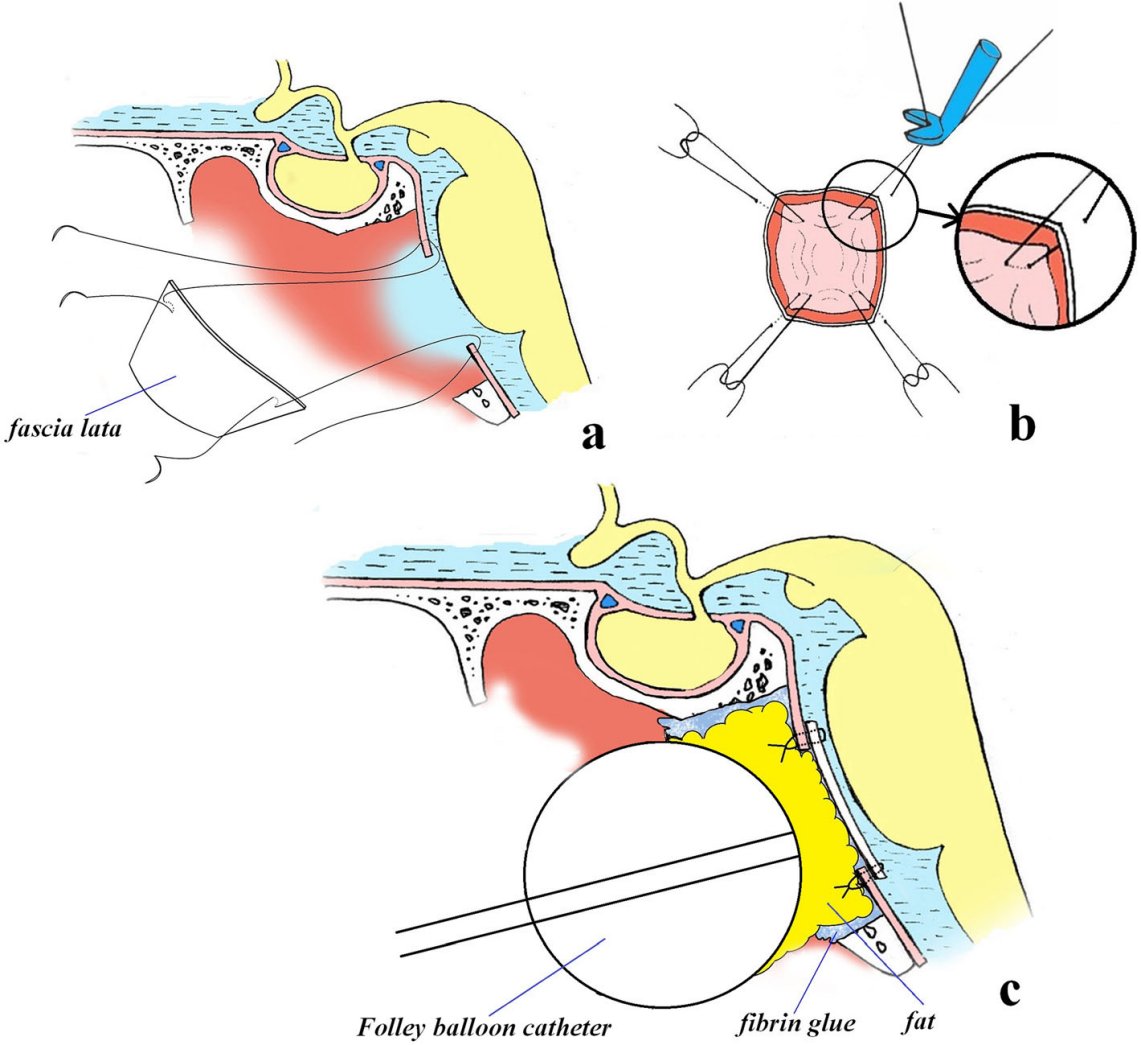
Skull base reconstruction technique using fascia lata, fixed to the dura mater with microsutures with an overlap. **a** Initial reconstruction stage; the autograft is fixed 2–3 mm from the edge. **b** Fixation of the free autograft at the corners and its lowering into the dura mater defect. **c** Appearance after reconstruction

In cases when the dura mater edges could not be approximated (for example, after extensive cautery or resection of the dura), the reconstruction technique involved fascia lata fixation to the dura mater edges using microsutures along the perimeter of the defect (Fig. 7). Another technique, involving fascia lata fixation to the dura mater using microsutures with an overlap was also used (Fig. 8).

To reduce the risk of cranial nerve dysfunction in the postoperative period, over the last 3 years, we have developed and introduced into clinical practice a novel method of intraoperative endoscopic neuromonitoring of the cranial nerves [22–24]. When removing tumors of the clivus (using the endoscopic transclival approach), intraoperative CN neuromonitoring was utilized in 11 cases (21 cranial nerves were identified intraoperatively).

**Fig. 9:**
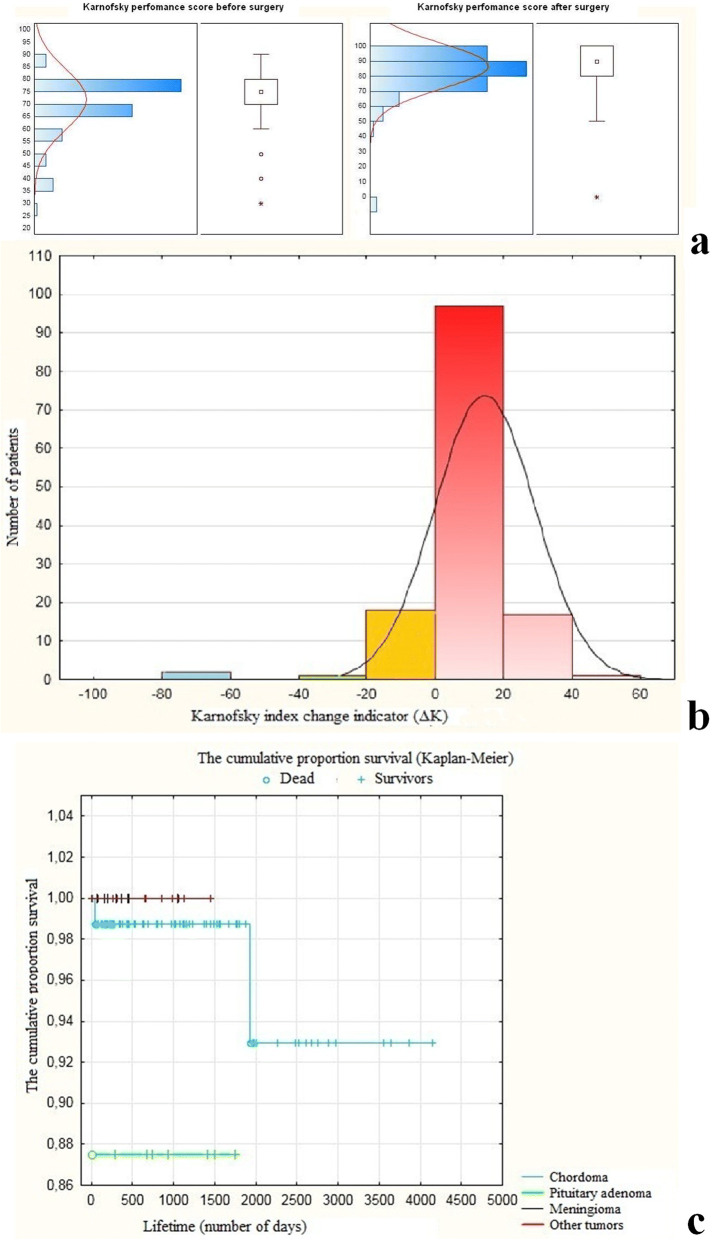
Statistical characteristics of the Karnofsky Performance Status Scale values and the patient survival rate after surgery. **a** Karnofsky Performance Scores in the study group before and after surgery (*n* = 140). (White square—the thick line segment is median; white circle—outliers; asterisk—extremes). **b** Histogram reflecting the distribution of the Karnofsky index change indicator among the patients (Δ*K* = *K*_after surgery_ − *K*_before surgery_), where *K* = Karnofsky index. **c** Patient survival rate (Kaplan-Meier). The 3-year survival rate of patients with chordomas was 98.3%, while the 5-year survival rate amounted to 92.3%. The 3- and 5-year survival rate in the group with pituitary adenomas was 87%. The 3-year survival rate of patients with meningiomas and other tumors was identical and amounted to 100%. In this study, the confidence interval was 95%, and the statistical significance threshold was *p* < 0.05

Graphs showing the statistical characteristics of the Karnofsky Performance Status Scale values before surgery and their dynamics during the postoperative period (calculations were performed using Statistica 10 software) are presented in Fig. 9a. The functional impairment of the patients was, on average, more pronounced in the preoperative period than in the postoperative period (median score = 75, mean score = 71.69, score range = 60, interquartile range = 10). In the postoperative period, the Karnofsky Performance Score distribution characteristics among these patients were as follows: median score = 90, mean score = 86.18, and interquartile range = 10. The results demonstrate a statistically significant difference between the average Karnofsky Performance Scores in the preoperative period and after surgery (71.69 and 86.18, respectively). In this study, the confidence interval is 95% with the critical level of significance at *p* = 0.05.

Figure 9b demonstrates the results of the statistical analysis of the dynamics of the Karnofsky index changes in the form of a histogram, with the confidence interval of 99% and a significance threshold at *p* < 0.01. The histogram displays the absolute value of the difference (Δ*K*) between the values of the Karnofsky index after and before surgery (Δ*K* = *K*_after surgery_ − *K*_before surgery,_ where *K* = Karnofsky index). Red color on the graph represents the number of cases (patients), associated with a positive Karnofsky index change (Δ*K* > 0), (*n* = 120, 85.714%). No positive Karnofsky index changes (Δ*K* = 0) were observed in 17 patients, representing 12.142% of the total number of patients (yellow column on the graph). Negative changes in the Karnofsky index (Δ*K* < 0) are represented by the green column on the graph (*n* = 1, 0.714%). The blue column represents the cases with a lethal outcome (*n* = 2, 1.43%).

For the majority of the patients (*n* = 105, 75%), change in the Karnofsky index ranged between 10 and 20 points. For 10.71% (*n* = 15) of the patients, change in the Karnofsky index ranged between 30 and 60 points. The average value of the Karnofsky index change was 14.18 points with an average postoperative score of 85.91. It should be noted that in 27.86% (*n* = 39) of the patients, the postoperative Karnofsky index amounted to 100, which indicates a significant improvement in the patient’s health status.

Tumor growth relapsed in 17 patients (15 patients with chordomas, 1 patient with a meningioma, and 1 with fibrous dysplasia), which led to reoperation over a period ranging from 17 months to 10 years after the first surgery.

Figure 9c presents data on the rate of patient survival (depending on the histological nature of the tumor).

## Discussion

Numerous variations of the craniotomy procedure have been developed to achieve minimal invasiveness. There are several types of retrosigmoid approaches, for example, which were designed for different tumor locations: the upper, middle, or lower neurovascular complexes of the cerebellopontine angle [28].

The endoscopic endonasal transsphenoidal approach (EETA) enables effective surgical treatment of tumors with maximum preservation of the skull base integrity. The endoscope facilitates manipulations associated with the removal of the tendons of the longus capitis muscle, trepanation of the clivus, and control of bleeding from the venous plexuses adjacent to the clivus. Wide trepanation of the clivus and large defects of the dura mater increase the risk of postoperative cerebrospinal fluid leakage. For the purposes of skull base reconstruction and reduction of the risks of CSF leakage, various techniques have been devised, including the use of balloon catheters, nasoseptal flaps, and microsurgical duraplasty. When utilizing these modern techniques, the risk of postoperative cerebrospinal fluid leakage can be diminished to 0–9.5% [29–31].

Understanding the relationship between the extent of the involvement of the clivus in the pathological process and the intradural neurovascular complexes is extremely important. Chordomas, for example, demonstrate intradural extension in more than 50% of cases. Other intradural tumors of the anterior central region, such as epidermoid cysts, neurenteric cysts, meningiomas, and cavernous malformations of the brain stem, can be surgically treated using the endoscopic transclival approach [32]. The transclival approach can also be used for the clipping of centrally located posterior cranial fossa aneurysms, granted the aneurysm can be clipped using the transcranial approach or embolized endovascularly. Aneurysms originating from the superior cerebellar artery (SCA), anterior inferior cerebellar artery (AICA), or posterior inferior cerebellar artery (PICA) can be surgically treated using the upper, middle, and lower transclival approaches, respectively [32].

The EETA has an advantage in terms of providing optimal exposure and a direct view of the midline structures. However, the use of the transnasal approach can also be associated with the risk of damaging the lateral parts of the cranial nerves (oculomotor, abducent, glossopharyngeal, vagus). The EETA can be considered as an independent and universal approach to pathological lesions of the skull base, and the choice of the approach should be based on the location of the tumor (especially regarding its relation to the cranial nerves) [33].

With the advent of extended endoscopic approaches in skull base surgery, 360° visualization when combined with other transcranial approaches has become a possibility. Selection of a specific approach depends on the anatomical and clinical characteristics of an individual patient, as well as the level of a surgeon’s proficiency in using endoscopic approaches [33, 34].

Compared with transcranial approaches to the base of the skull, the EETА has a number of advantages, which can reduce the rate of complications, associated with open access surgery:

1. There is no need to perform traction on various structures of the brain.
2. A wider angle of view.
3. No need to displace the vertebral artery.

The EETА also has some advantages compared to transcranial approaches:

1. As a fully endoscopic procedure, it requires no traction of brain structures.
2. The approach allows for an extended visualization of the extra-intradural space from the crista galli to the craniovertebral junction.
3. The approach provides a well-lit surgical corridor and, consequently, adequate visualization of even the most inaccessible regions [2–4, 11].

Since the endonasal surgical corridor is not associated with traumatization of the structures of the oropharynx and the soft palate, it significantly reduces the risk of bacterial contamination and infection. In addition, the patients have a low risk of postoperative swallowing and speech disorders and are capable of oral food intake without risk of dysphagia immediately after surgery.

The EETA can be difficult to perform in cases of atypical topography of the neurovascular structures located medially and anteriorly in relation to the tumor. Relative contraindication for this approach is a significant lateral displacement of the tumor at the level of the foramen magnum posterior to the occipital condyle, as there is a risk of craniocervical instability and injury to the caudal group of nerves [35]. The EETA can be effectively used for removing tumors located centrally in the skull base and extending into the posterior cranial fossa.

Reconstruction of the skull base defect after tumor removal is one of the main stages of the surgery. We used different reconstruction techniques, and in our view, autograft fixation using microsutures is preferable and more reliable, even though it requires additional training and is technically more demanding, leading to longer surgery duration and slightly higher postoperative complication risks.

The separation of the clivus into the upper, middle, and lower sections by the transverse lines located at the level of the dural openings of the abducent and glossopharyngeal nerves is based on the concept of three neurovascular complexes in the posterior cranial fossa [2, 32]. Approaches to the upper, middle, and lower sections of the clivus provide access to the anteromedial region of the three mentioned neurovascular complexes [1, 32, 36]. The extended approach to the upper section of the clivus provides access to the midbrain, the upper half of the pons, the superior cerebellar artery, and the oculomotor and trigeminal nerves in the upper neurovascular complex. Approaches to the middle section of the clivus provide access to the lower half of the pons, the anterior inferior cerebellar artery, and the abducent, facial, and vestibulocochlear nerves in the middle neurovascular complex. Approaches to the lower section of the clivus expose the medulla oblongata, the posterior inferior cerebellar artery, and the glossopharyngeal, vagus, accessory, and hypoglossal nerves in the lower neurovascular complex.

## Conclusion

Median tumors of the skull base are extremely difficult targets for surgical treatment using the standard transcranial approaches. Previously, patients with tumors of this localization mostly underwent palliative surgery: bypass surgery to resolve hydrocephalus, decompression of the posterior cranial fossa structures, or partial tumor removal using standard transcranial approaches. The endoscopic endonasal posterior extended (transclival) approach, as a minimally invasive procedure, allows for surgical removal of various tumors of the central region of the skull base involving the clivus, which, until recently, were considered difficult or impossible to access for surgical treatment. Tumor removal using the endoscopic endonasal transclival approach is characterized by high radicality, low risk of postoperative complications, and low mortality [37, 38]. These technological surgical interventions must be carried out in highly specialized hospitals, where neurosurgeons have extensive experience using both endoscopic and microsurgical techniques on various structures of the skull base.

## Abbreviations

AICA: Anterior inferior cerebellar artery
CN: Cranial nerve
CSF: Cerebrospinal fluid
CT: Computed tomography
EETA: Endoscopic endonasal transsphenoidal approach
ICA: Internal carotid artery
MRI: Magnetic resonance imaging
PCA: Posterior cerebral artery
PICA: Posterior inferior cerebellar artery
SCА: Superior cerebellar artery
